# A longitudinal study examining the effect of carer-child relationship quality on child’s emotional and behavioural difficulties while in care

**DOI:** 10.1007/s00787-025-02900-9

**Published:** 2025-11-10

**Authors:** Rosa Sparks, Jala Rizeq, Karen Crawford, Helen Minnis

**Affiliations:** 1 School of Health and Wellbeing, University of Glasgow, 90 Byres Road, Glasgow, UK; 2 Gillberg Neuropsychiatry Centre, University of Gothenburg, Gothenburg, Sweden

**Keywords:** Care experience, Placement instability, Mental health, Carer-child relationship quality

## Abstract

Early relationships between a caregiver and their child set the foundations for many aspects of the child’s development. Exposure to abuse or neglect can negatively impact the security and stability within these early relationships. The aim of this project was to characterise the association between carer-child relationship quality and child’s mental health over a period of 2.5 years. We used data collected between 2011 and 2022 as part of the Best Services Trial (BeST^?^). The sample consisted of 220 children entering foster/kinship care who were between 0.08 (0.96 months) and 5.58 years at first data collection visit. Relationship quality was assessed using Parent-Infant Relationship Global Assessment Scale (PIR-GAS), and child emotional and behavioural difficulties was assessed using the Strengths and Difficulties Questionnaire (SDQ) and The Infant Toddler Social Emotional Assessment (ITSEA). Data was examined from two time points, a few weeks after entering foster/kinship care and after 2.5 years. The quality of the carer-child relationship did not predict emotional and behavioural outcomes for the child at a later time point. Nonetheless, we found that overall, children had significantly better relationships with their carers at the 2.5 year follow up than at baseline. This highlights a need for specific tailored interventions for care-experienced children, to support their emotional and behavioural needs and improve long term outcomes.

## Introduction

In 2021/2022 there were approximately 105,400 children in care in the United Kingdom, and this number is growing with an increase of 9% in the past five years [1]. This number includes children who have been placed in foster care, kinship care, those who remain at home with their parents (Scotland) and those in residential accommodation. Due to different legal systems in Scotland, many children who are in the care system continue to live at home with their parents but are under a supervision requirement, which requires them to have regular contact with social services [2]. Although reasons for entry to care vary, exposure to adversity including abuse or neglect is commonly reported in care-experienced children and young people [3]. These experiences are often referred to as adverse childhood experiences (ACEs) which is a term used to categorise stressful events that occur in childhood, such as exposure to domestic abuse, having a parent with a mental health condition, a household member being in prison and exposure to abuse and neglect [4]. A large cohort study conducted in the USA found that 40.8% of children and young people in care reported between six and nine ACEs, and 37.4% reported more than ten ACEs [5]. Therefore, this early exposure to adversity can have an impact on how children go on to form relationships with their caregivers, considering children’s feelings of safety and security are being disrupted by the maltreatment [6]. ACEs not only impact how children form relationships, but they also increase the risk of poor mental and physical health throughout childhood and the lifespan [7] Racine and colleagues (2020) found that cumulative childhood adversity predicted the level of trauma related distress a child experiences. However, they also found that children who had high levels of protective factors (such as coping strategies, peer support and caregiver physical and psychological caregiving) were protected from this [8]. This suggests that trauma related symptoms can be mitigated by bolstering a child’s protective factors, which includes a positive relationship with a caregiver.

The caregiver-child relationship is the first crucial relationship that a child forms, and the quality of this relationship impacts many aspects of the child’s development [9]. Based on attachment theory, the security of the early parent child relationship impacts how the child forms interpersonal relationships throughout the course of their life [10, 11]. According to Ainsworth (1979), this early attachment to one’s caregiver can shape how one goes on to relate not only to their caregiver but also to other people and objects in their environment, underlying the child’s future social and emotional development, even as they gain new cognitive and interpersonal skills [10]. A secure attachment forms when a child perceives their caregiver to be responsive and sensitive to their needs and offers a safe base for them to explore the world from [12]. A lack of an early secure attachment can impact on a child’s mental health, with reviews of the literature finding that insecure attachment is associated with higher levels of anxiety and internalising difficulties compared to secure attachments [13, 14]. Early exposure to adversity and caregiver disruption hinders the development of secure attachments [15]. A meta-analysis examining the attachment of pre-school aged children in foster care, found that approximately 40% of children in care had insecure attachments and approximately 22% of children had disorganised attachment [16].

Children in care are also at risk of developing attachment disorders, such as reactive attachment disorder (RAD) [6, 17]. Children with RAD show disturbances in how they interact with others, and this is characterised by lack of attachment behaviours (e.g., reaching out to be picked up), focused towards their caregiver(s). Children with RAD display these disturbances in attachment behaviours with different individuals and across different contexts rather than just with the primary caregiver [18]. Risk factors for RAD include; neglect, physical abuse, sexual abuse, parental alcoholism, parental mental illness, parental drug use, and the absence of a consistent primary caregiver [19], commonly present in children who have entered into care. These difficulties may therefore impact how they interact with their carer and in turn pose challenges on the quality of the child-carer relationship.

Due to these early attachment disruptions as well as high levels of adversity often experienced by children in care, this population group is especially at risk for developing mental health difficulties. Compared to the general population, it has been estimated that children in care are three times more likely to have a mental health disorder than in children who have not been in care [20]. These numbers are high, with one study finding that 60.5% of pre-school aged children who are in care have a diagnosis of at least one mental health disorder [21]. The most common diagnoses of children in care were found to be major depressive disorder, oppositional defiant disorder, conduct disorder, reactive attachment disorder and post-traumatic stress disorder [22]. Levels of externalising difficulties (such as hyperactivity or behavioural difficulties) are also common, with one study finding 40.6% of a sample of children in care having externalising difficulties [23].

Another factor which has been found to impact the mental health of children in care is the number of care placements they experience. Throughout their time in care, children may experience multiple placement changes, with 10% of children in care in England and 4% of children in care in Scotland experiencing three or more placement moves within a 12-month period [1, 2]. This instability in residence also represents an instability in caregiving which could lead to children struggling to develop meaningful relationships with caregivers. There is a significant body of literature exploring the impact of placement instability on emotional and behavioural difficulties of children in care, which shows that placement instability is associated with poorer outcomes [24–26]. Importantly, stable care placements are associated with positive emotional and behavioural outcomes for children [27–30]. Therefore, placement instability is an important factor to consider when exploring the impact of the carer-child relationship on mental health outcomes for care-experienced children and young people. This study aimed to explore whether the carer-child relationship predicts emotional and behavioural difficulties, while controlling for placement stability/instability.

### The current study and aims

The current evidence base highlights the importance of a positive carer-child relationship, however this relationship has not been examined longitudinally for children who are in care, especially in early childhood. There is also a lack of research which explores the association between the carer-child relationship and child mental health outcomes, and it is unclear how these two domains impact on one another, above and beyond the impact of number of placements a child has experienced. Therefore, the primary aim of this project was to characterise the strength and direction of the association between carer-child relationship quality and child’s emotional and behavioural difficulties over a period 2.5 years in care. All children in this study were entering foster care or kinship care at the beginning of the trial, however over the course of the 2.5 years some of these children may have returned home to their birth parents or were adopted. Therefore ‘carer-child relationship’ refers to either foster/kinship carer and child or birth/adoptive parent and child. A secondary aim was to understand the unique effects of carer-child relationship quality on child’s emotional and behavioural difficulties, above and beyond the effects of relevant factors including child sex, age at entry to care, and number of placement moves.

**Research Questions**.

1. What is the direction of the association between carer-child relationship and child emotional and behavioural difficulties over a time period of 2.5 years?
2. Does the quality of the carer-child relationship predict the child’s emotional and behavioural difficulties at a later time point, above and beyond the effect of number of placement moves?

## Method

### Design and procedure

This study used a longitudinal repeated measures design, using existing data collected between 2011 and 2022 as part of the Best Services Trial (BeST^?^) [31]. BeST^?^ is a randomised control trial comparing an infant mental health service (the New Orleans Intervention Model (NMI) with the Social Work Services (as usual). The aim of this trial was to explore what the best service is for abused and neglected pre-school aged children coming into foster care. Families were recruited into the trial if they had a child aged between 0 and 5 years entering an episode of foster or kinship care due to reasons associated with maltreatment, in either of the trial sites (Glasgow or London). Participants were included in the trial upon completion of written informed consent from the birth family and the child’s foster or kinship family. Participants completed quantitative measures over a period of 2.5 years. Measures of child mental health and carer-child relationship functioning were collected at three time points throughout the trial; (1) a few weeks after entering foster care (T1), (2) 15 months after entering care (T2) and (3) 2.5 years after entering care (T3). To be eligible for inclusion in this current study, participants had to have completed a baseline rating of the quality carer-child relationship at T1. Because T3 was the end date and primary outcome point for the trial and due to disruptions with data collection at T2 due to COVID-19 restrictions, only T1 (baseline) and T3 (end of trial) data when available were included in the current study.

To complete the assessments, carers were invited to health care settings where they completed a number of questionnaires and a video recording was taken to observe interactions which was used to score the quality of the relationship. Due to the COVID-19 pandemic and lockdown restrictions, some carers were asked to complete these measures and video recordings at home. During and post COVID-19, questionnaire measures were taken by telephone in the majority of cases, with a small number of respondents opting for self-completion by post. Where video data was captured (for PIRGAS: see measures section below) respondents were asked to replicate the play and snack scenario protocol as were used in clinic visits. They would then record using their own devices and upload the video data via a link to the university’s secure data transfer platform. Videos were then scored by the rater team at a later date. Overall the type of visit was coded as either face to face or remote (T1: face to face *n* = 183, remote *n* = 25, not recorded *n* = 12; T3: face to face *n* = 122, remote *n* = 52, not recorded *n* = 46).

The BeST^?^ Trial was approved by the West of Scotland Research Ethics Service, Committee 3 (approval number 15/WS/0280) and this current study was covered within the project’s ethical approval [31]. Informed written consent was obtained from all carers and each child (participant) in the study was assigned a unique ID number to link their data throughout the trial in order to maintain confidentiality.

## Participants

The total number of participants recruited to the BeST^?^ was 488 children, from 382 families, 378 of whom were in Glasgow and 110 in London. Of this sample, data from 220 participants who had a rating of the quality carer-child relationship at T1 were included in this study. Of those, 101 participants had complete data across T1 and T3. The age range of the participants was between 0.08 and 5.58 years. The sample characteristics of the 220 participants included are summarised in Table 1. Of the 220, there were 1.82% without a recorded ethnicity and 0.45% without a recorded index of multiple deprivation.

**Table 1:** Sample characteristics

Demographics		%
Sex	Male	53.64%
	Female	46.36%
Ethnicity	White	86.82%
	Mixed	5.00%
	Asian/Asian British	4.09%
	Black/Black British	2.27%
Supervision order	Compulsory	52.73%
	Voluntary	47.27%
Index of Multiple Deprivation (IMD) decile	1 (most deprived)	63.18%
	2	18.64%
	3	9.09%
	4	5.45%
	5	2.27%
	6	0.91%

### Materials and measures

#### Child emotional and behavioural difficulties

Depending on child age, emotional and behavioural difficulties were measured using one of two or both measures: the Strengths and Difficulties Questionnaire (SDQ) [32], and the Infant Toddler Social Emotional Assessment (ITSEA) [33] described below.

SDQ is a measure which is used to assess child’s mental health in children aged 2–17 years old [32]. The 25-item carer-report version of the SDQ was used in this study. The version used with two to four year olds has three items modified from the original four to 17 year old version, to ensure developmental appropriateness [34]. Both versions of the SDQ were used in this trial (two- to four-year-olds and four- to 17-year-olds). The SDQ has 5 subscales; emotional symptoms subscale, conduct problems, hyperactivity/inattention, peer relationship problems and prosocial behaviour. These are scored on a three-point scale; not true, somewhat true and certainly true. Somewhat true is always scored as a 1, but the score for not true and certainly true varies between the item (either scored as 0 or 2). The scores from all the subscales, excluding prosocial behaviour, add together to generate a total difficulties score, which was the score used in this study, with higher scores indicative of more difficulties. The SDQ has been shown to have good reliability and validity [35].

For children under the age of 2, the ITSEA was used [33]. This is a carer-report measure used to assess social-emotional and behavioural problems. It consists of 166 items across 4 domains; externalising (activity/impulsivity, aggression/defiance and peer aggression), internalising (depression/withdrawal, general anxiety, separation distress and inhibition to novelty), dysregulation (negative emotionality, sleep and eating problems, sensory sensitivity) and competence (attention, mastery, motivation, play, empathy and prosocial peer relations). Items are rated as 0 = not true/rarely true, 1 = somewhat true/sometimes and 2 = very true/always. For some items there is an option to respond ‘N’, which would be used where the parent or carer has not had the opportunity to observe that behaviour. In these instances, items marked ‘N’ are scored as missing or ‘M’ and if there are two or more ‘M’ answers in each subscale the subscale cannot be used. For this study the ITSEA was scored in alignment with the manual. The ISTEA has been found to be a valid measure to assess emotional and behaviour problems in children under 2 years [36].

Total emotional and behavioural difficulties score was computed using either the SDQ or ITSEA, depending on availability, and converted into a standardised z score to allow comparability. For the ITSEA an average of both the internalising and externalising z-scores represented total emotional and behavioural difficulties score. For the SDQ the total difficulties score was converted into a z score. Where there was both an SDQ score and an ITSEA score available for one child, the SDQ score was prioritised and used.

#### Carer-child relationship functioning

The Parent Infant Relationship Global Assessment Scale (PIR-GAS) [37] was used to assess carer-child relationship quality as indicated by Diagnostic Classification of Mental Health and Developmental Disorders of Infancy and Early Childhood (DC:0–3). The PIR-GAS is commonly used as a rating instrument and is used in clinical settings to observe parent-infant relationships, to describe the strengths of a relationship as well as the severity of a disorder. PIR-GAS can also be used as a research tool [38]. Three aspects of the relationship are evaluated: *behavioural quality of interactions*,* affective tone*, and *psychological involvement.* The relationship is initially placed into one of ten categories ranging from well adapted (100 − 91) to grossly impaired (10 and under), therefore a higher score would indicate a better quality relationship. A continuous measure of carer and child relationship quality was created by choosing a final score within the chosen decile.

PIR-GAS scores were derived from video recording of the carer child interaction. Carers were asked to play with their child for a period of time and eat lunch with their child and both elements were recorded. The videos were rated by a team of trained raters, including the primary author, and scored using the PIR-GAS rating scale. 20% of the videos were rated by a second rater to establish inter-rater reliability. Where there was a discrepancy of more than 10 points, scores were discussed at conference with experienced PIR-GAS raters, including the research supervisor, and a final score was agreed.

#### Placement instability

To explore placement instability each child experienced prior to the trial, a binary variable was created. This was coded as 0 or 1, where 0 was no prior placement moves and 1 was one or more placement moves. In total 174 (79%) children had experienced 0 prior placement moves, 22 (10%) had experienced 1 placement move and 2 (1%) had experienced 2 or more placement moves. There is missing data for 22 (10%) children.

#### Index of multiple deprivation (IMD)

The IMD measures deprivation across nations of the United Kingdom. Postcodes of parents’ addresses at time child came into care were collected and used to assign decile value in respect to level of deprivation. Areas are ranked from the most deprived (rank 1) to least deprived area (rank 10). The observed range in our sample was 1 to 6. Each area measures deprivation in a slightly different way, however, mostly considering income, education, employment, crime and living environment [39]. The IMD was used to further characterise the sample demographics.

### Analytic strategy

Analyses were conducted using R Studio (R version 4.3.3). Preliminary screening of the data was conducted using descriptive statistics and visualised using histograms and scatterplots to assess normality (univariate distributions) and linearity (bivariate distributions), respectively. To examine the associations between carer-child relationship quality and child emotional and behavioural difficulties at the two time points, bivariate Pearson correlations were estimated.

To examine if the quality of the carer-child relationship at T1 predicted child emotional and behavioural difficulties at T3, above and beyond the number of previous placements and whilst controlling for child initial emotional and behavioural difficulties, multiple regression analysis was used. Two regression models were estimated, firstly using PIR-GAS score at T1 as the predictor and next using a PIR-GAS change score as the predictor (the difference between T1 PIR-GAS score and T3 PIR-GAS score).

To explore the longitudinal association between carer and child relationship quality and child emotional and behavioural difficulties and the direction of this relationship, a cross-lagged path analysis model was estimated. This model was estimated using the lavaan package (version 0.6–7; [40] and adjusted for multivariate non-normality using maximum likelihood estimation with robust standard errors and fit statistics [50]. Missing ratings were handled using full-information maximum likelihood. The model estimated was fully saturated and therefore model fit was not assessed. A sensitivity analysis was conducted, estimating the same cross-lagged panel model, using data from the subset of participants who have complete data across T1 and T3.

### Sample size

To ensure the current study would be well powered, a sample size analysis was calculated prior to data analysis. This was completed based on previous effect taken from research with the same sample using the SDQ scores at time 3 and carer commitment scores at time 1. With an effect size of 0.201, in a multiple regression with 4 predictors using a power of 0.8 and an alpha level of 0.05, it was estimated we would need a sample of 66 participants. For the path analysis model, our sample being between 100 and 200 is considered acceptable [41]. Our path analysis includes 8 free parameters, and as such a sample of 220 (and a sample of 101 with only complete data) is well powered to conduct the analysis.

## Results

### Descriptives and correlations

In our sample, there were 211 children from Glasgow and 9 from London^1^. Table 2 presents the descriptive statistics for the variables included in this study (*n* = 220) and shows the degree of missingness across variables and timepoints. Of the total sample, 53.64% were Male and 46.36% Female. The majority of the sample were White (86.82%) and 63.18% were from the most deprived areas according to the IMD. The mean age of the sample was 2.44 years old. In terms of the missingness of data, the full sample of 220 participants had PIR-GAS at T 1, and 101 at T3, and 146 had an SDQ/ITSEA at T1 and 188 at T3. Table 3 shows the bivariate correlations. Overall, there was no meaningful correlation between the quality of the carer-child relationship (PIR-GAS score) and the child’s emotional and behavioural difficulty score at T1 and T3; all associations were non-significant and small. Emotional and behavioural difficulties at T1 showed a small-moderate and significant correlation with emotional and behavioural difficulties at T3 (*r* = 0.30, *p*< 0.05). There was a small-moderate negative correlation between placement instability and carer-child relationship quality at T3 (*r* = −0.34, *p* < 0.05), indicating instability was associated with poorer relationship quality at T3. Placement instability also had a small significant association with emotional and behavioural difficulties at T3 (*r* = 0.19, *p* < 0.05). Sex and carer-child relationship quality at T1 showed a weak but significant correlation (*r* = 0.20, *p* < 0.05), indicating that higher quality relationship is reported with females as compared to males. Age at T1 showed a weak but significant correlation with carer-child relationship quality at T1 (*r* = 0.23, *p* < 0.05) and emotional and behavioural difficulties at T3 (*r* = 0.18, *p* < 0.05), indicating that older age at entry to care is associated with higher relationship quality ratings at T1 and higher emotional and behavioural difficulties at T3.

**Table 2:** Descriptives of variables

Variable	*N*	Mean	SD	Min	Max
Age (T1)	220	2.44	1.58	0.08	5.58
Carer-child relationship quality T1	220	80.07	13.58	25	100
Carer-child relationship quality T3	101	85	10.58	35	98
Emotional & behavioural difficulty T1	146	−0.02	0.91	− 1.52	2.60
Emotional & behavioural difficulty T3	188	0.00	1.00	−1.55	2.67
SDQ score T1	102	12.20	8.01	0.00	33
SDQ score T3	188	11.36	7.35	0.00	31
ITSEA internalising score T1	69	0.47	0.25	0.00	1.06
ITSEA externalising score T1	69	0.61	0.41	0.00	1.58
ITSEA internalising score T3	5	0.45	0.30	0.10	0.76
ITSEA externalising score T3	5	0.30	0.22	0.00	0.53

**Table 3:** Correlations among variables

Variable	1	2	3	4	5		6		7
1. Carer-child relationship quality T1	1								
2. Carer-child relationship quality T3	0.10	1							
3. Emotional & behavioural difficulties T1	−0.09	0.04	1						
4. Emotional & behavioural difficulties T3	−0.06	−0.10	0.30*	1					
5.Placement instability	0.05	−0.34*	0.04	0.19*	1				
6. Sex	0.20*	0.00	−0.04	−0.06	0.05		1		
7. Age T1	0.23*	0.05	−0.03	0.18*	0.05		−0.06		1

Note *significant at *p* < 0.05

### Multiple regression

Table 4 summarises the results from the multiple regression analysis with emotional and behavioural difficulties at T3 as the outcome. The first model uses carer-child relationship quality score at T1 as the predictor and the second model uses carer-child relationship quality change score as the predictor (calculated by subtracting PIR-GAS score at T1 from PIR-GAS score at T3). Model 1 (F(5,108) = 2.40, *p* = 0.04) explained 10% of the variance, with an adjusted R² of 0.06. Model 2 (F(5,68) = 1.91, *p* = 0.10) explained 12.3% of the variance, with an adjusted R² of 0.06. In both models, only child emotional and behavioural difficulties at T1 was a significant predictor of the outcome at T3. None of the other predictors had a significant effect, meaning that the quality of carer-child relationship at T1, or the change in this relationship, did not predict child emotional and behavioural difficulties at T3 above and beyond other variables considered, which also did not present with any significant effects on the outcome.

**Table 4:** Multiple regression models examining unique effects on child emotional & behavioural difficulties at T3

Model 1	b	β	t	*p*
Carer-child relationship quality score T1	−0.00	−0.06	−0.63	0.53
Placement instability	0.21	0.08	0.84	0.40
Sex	−0.07	−0.03	−0.35	0.73
Age at T1	0.10	0.12	1.33	0.19
Child emotional & behavioural difficulties T1	0.28*	0.28	3.04	< 0.01
*Model 2*				
Carer-child relationship quality change score	−0.00	−0.02	−0.14	0.89
Placement instability	0.18	0.06	0.46	0.65
Sex	−0.02	−0.01	−0.10	0.92
Age at T1	0.07	0.10	0.83	0.41
Child emotional & behavioural difficulties T1	0.32*	0.35	3.05	< 0.01
Note *significant at *p* < 0.01				

### Cross lagged panel model

**Fig. 1:**
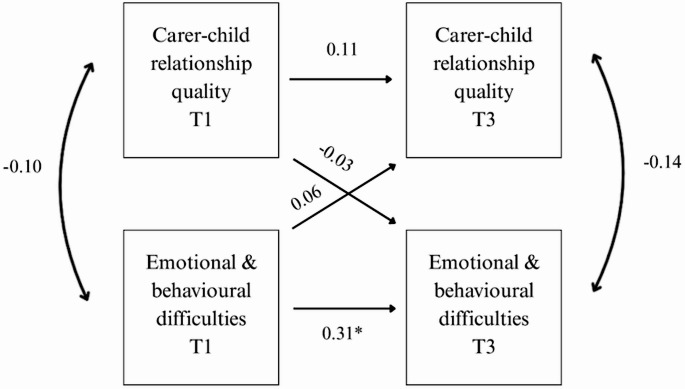
Path Analysis Model *Note *Figure 1 shows the standardised regression coefficients of the stability and the cross lagged effects. It also shows the correlation coefficients between variables at the same time point (double headed arrows). *indicates a p value of less than 0.05

**Fig. 2:**
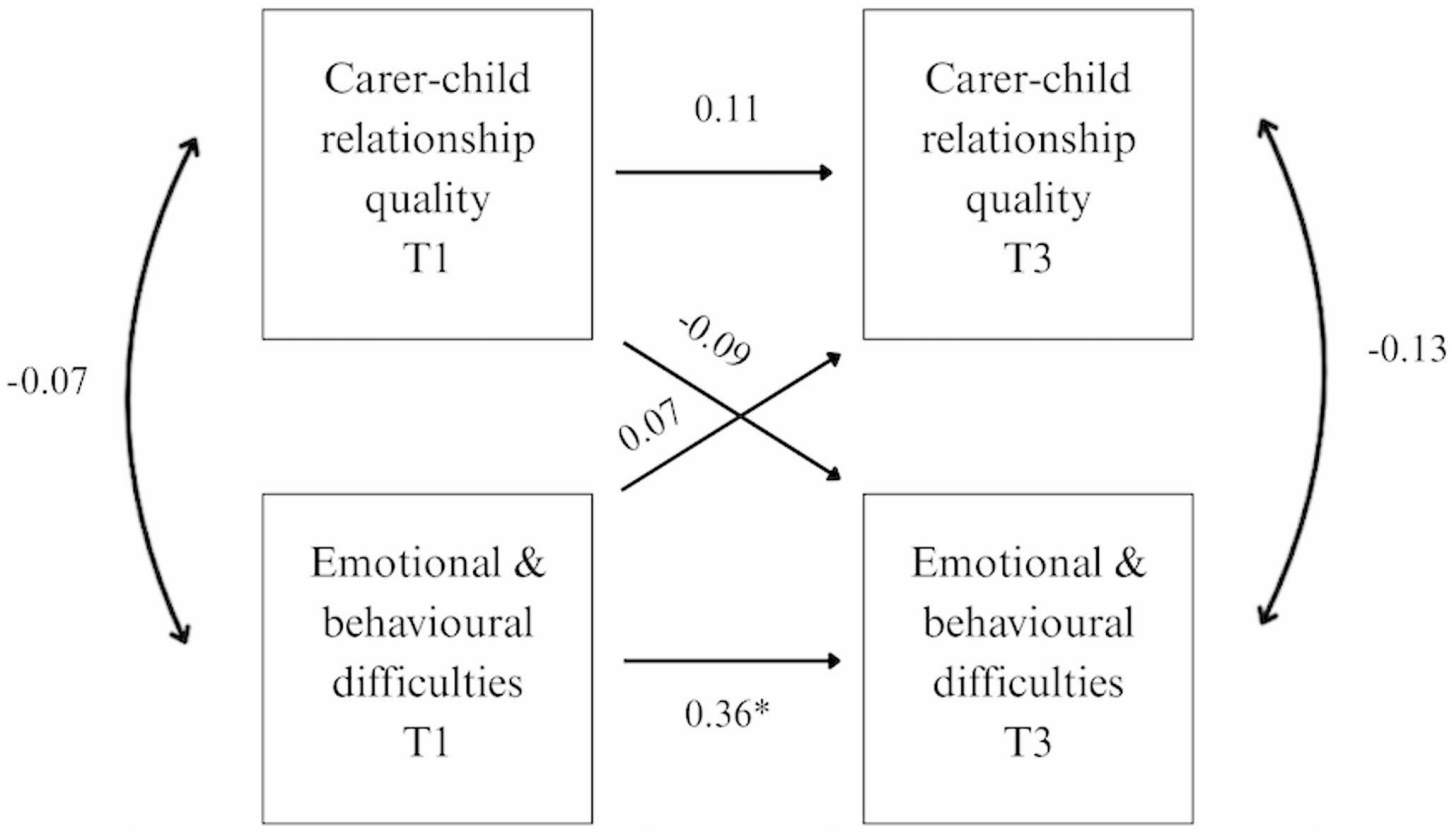
Path Analysis Model for the Sensitivity Analysis *Note *Figure 2 shows the standardised regression coefficients of the stability and the cross lagged effects. It also shows the correlation coefficients between variables at the same time point (double headed arrows). *indicates a p value of less than 0.05

Figure 1 below shows the cross lagged panel model, depicting the direction of the paths between carer-child relationship quality score and child emotional and behavioural difficulties score over time. The figure includes the standardised regression coefficients. As shown in Fig. 1, there is no stability in carer-child relationship quality scores from T1 to T3 (*β* = 0.11, *p* = 0.200). There was some stability in emotional and behavioural difficulties scores from T1 to T3 (*β* = 0.31, *p* = 0.002). None of the cross lagged effects were significant, and both very small. The model explained 10% of the variance in emotional and behavioural difficulties at T3 and 1.5% of the variance in carer-child relationship quality scores at T3. The sensitivity analysis (Fig. 2), using only complete data across T1 and T3 (*n* = 101), yielded similar results.

Based on these findings, further exploratory analysis was completed to examine the differences in carer-child relationship quality and emotional and behavioural difficulty scores between T1 and T3. The distribution of change scores were assessed by visually examining the histogram, with no issues noted with normality. Two paired samples t-tests were performed to compare the difference in carer-child relationship quality scores and in emotional and behavioural difficulties between T1 and T3. There was a significant difference, with higher carer-child relationship quality scores at T3 (*M* = 85.00 *SD* = 10.58) than at T1 (*M* = 80.07 *SD* = 13.58), t(100) = 1.99, *p* = 0.0495 with a 95% mean difference confidence interval = 0.01, 6.09. The effect size as measured by Cohen’s d was d = 0.31, indicating a small effect. The findings indicate that the overall relationship quality between carer and child is rated as significantly higher at T3 compared to T1. In terms of emotional and behavioural difficulties, the t-test showed no significant difference between the emotional and behavioural difficulty scores at T1 (M = 0.02, SD = 0.91) and T3 (M = 0.00, SD = 1.00), t(125) = 0.86, *p* = 0.39, with a 95% mean difference confidence interval = -0.11, 0.28. The effect size as measured by Cohen’s d was d = 0.02, indicating a very small effect; this is consistent with the stability noted in earlier analysis.

## Discussion

The results of this study provide insight into how relationship quality between carer and child change over time spent in care, and the degree to which these relationships are implicated in the emotional and behavioural outcomes for the child. This study shows that the quality of the relationship between carer and child over time does not predict emotional and behavioural outcomes for the child, but the overall quality of this relationship is significantly stronger later in the child’s journey in care than early on.

### Placement instability

Consistent with literature highlighting the negative impact of placement instability on children’s mental health outcomes [21, 42], placement instability at the time of this study was significantly associated with poorer relationship quality and higher emotional and behavioural difficulties 2.5 years later. This is further evidence that the history of placement instability is a risk factor for negative outcomes. It is important to note that in this sample, the first entry to care for the majority of the children (88%) was at the time of entering this study. This could be due to the young age of the children at Time 1. Nonetheless, even at this young age, the presence of early instability had an effect on relationship quality and emotional and behavioural outcomes. The foster care system in the UK is unstable relative to that of other countries. In Scotland, many children experience repeated short-term episodes of foster care, specifically in Glasgow where two thirds of children who returned home re-entered the care system, often being referred to the ‘revolving door effect’ [43]. It is important to invest in supportive and stable placements for children to ensure we promote healthy development and functioning [44]. Relationship quality is a crucial factor in maintaining supportive and safe placements [45].

### Effect of relationship quality on emotional and behavioural outcomes

Based on the importance of the relationship quality between child and carer, it was expected that better quality of the relationship would be associated and predictive of lower emotional and behavioural difficulties for the child. However, the results from this study found that the quality of the child’s relationship with their foster carer did not predict later emotional and behavioural difficulties. Similarly, sex, age at entry to care and placement instability also did not predict later emotional and behavioural difficulties. The only predictor of later emotional and behavioural difficulties was baseline emotional and behavioural difficulties, with no differences in average difficulties between baseline and two and a half years into their time in care during the study.

This is an important finding to consider, as it may help to explain why some children placed in nurturing foster care do not see improvement in their emotional and behavioural functioning over time. Research has shown that stable mental health profiles are the most common in care-experienced children and young people and much less likely for children’s mental health trajectory to change over time in care [46]. Our findings show similar results. Another important consideration with children who have been abused and neglected is that as a group they are much more likely to have heritable neurodevelopmental conditions than their peers (not caused by abuse and neglect) [47]. Minnis [48] suggests that if the high rates of neurodevelopmental problems within this population are not due to the environment they are placed in, but more due to inherited difficulties, then it would not be expected that placement in foster care would ‘treat’ the difficulties, which again may speak to the stability of some of the emotional and behavioural difficulties measured in our study. This means that this group of children would require comprehensive mental health assessment and individualized treatment, and care placement alone, even when the quality of child-carer relationship is good or improves, is not sufficient to address emotional and behavioural difficulties.

### Stability in Carer-Child relationships

The lack of stability in the quality of the relationship between carer and child over time spent in care highlights the dynamic and evolving nature of relationships between foster carer and child. Nonetheless the relationship between carer and child improves over time spent in care, in part explaining the lack of stability. This is a hopeful finding for care-experienced children, as it shows the ability to improve a relationship with a caregiver over time spent in care, irrespective of their mental health and initial relationship quality. This suggests that whilst living in a nurturing foster care environment that provides stability, and given sufficient time, children are able to build connections with their caregiver and develop positive relationships. This is the first study we are aware of that has looked at this relationship over time using validated tools. It will be important to see how this relationship continues to change over a longer period of time in care, and for children at different developmental stages.

### Limitations and future directions

This is a novel study that examined how the quality of carer-child relationship changes over a period of time a child is in care and how this relates to child mental health, in a representative sample of young children in care. Although the sample size in this study was smaller than initially anticipated, due to missing data on the variables of interest, there was still sufficient information to answer the questions of interest. Further, attrition commonly occurs in longitudinal research, and in this study, it may also have been influenced by the COVID-19 pandemic, which occurred during the data collection period. The sensitivity analysis conducted using the subsample with complete data at Time 1 and 2.5 years later suggests that there is no bias resulting from attrition or missingness. Further, as reported in the main trial paper, there were no differences between those who completed follow ups from those who dropped out [49]. Additionally, the imbalance in the sample of children across the London and Glasgow sites introduces questions about representativeness and generalisability to contexts, especially that these two sites have different care systems and policies. Future research would benefit from process evaluation of the systems and their potential impact on outcomes. It would further be important for future research to build on these findings by exploring the impact that adversity has on this population. Increasing understanding of the impact of ACEs on the outcomes of care-experienced children will provide greater insight into how best to support their needs. Similarly, a greater understanding of the impact of neurodevelopmental conditions and their intersection with adversity would be valuable for future research to explore.

## Conclusions and implications

Overall, we found that the carer-child relationship ratings improved after 2.5 years in care. At the same time, this relationship did not predict emotional and behavioural outcomes. There are likely other factors which are influencing the emotional and behavioural difficulties of children in foster care, with a small effect observed due to initial placement instability. This highlights a need for further longitudinal research to better understand these relationships, and how they might support improvements in emotional and behavioural wellbeing of children in foster care. The results from this study show that placing a child in foster care and removing them from a neglectful and/or abusive home environment, does not, by itself, improve emotional and behavioural outcomes but that it can have positive effects on relationship functioning. This highlights the need for other measures to be put in place, including follow up assessments and the use of evidence-based treatment and management strategies which are tailored to care-experienced children, in order to improve the long-term outcomes for this population.[9, 15] 

## Data Availability

As the findings of this paper are based on data from a larger trial, all data requests will have to be submitted to the chief investigator (H.M.) and then will be reviewed and considered by the Trial Management Group (TMG).

## Footnotes

1 We conducted a Welch t-test to examine differences in the main outcome (emotional and behavioural difficulties) across the groups of children from Glasgow (Mean = 0.01, SD = 1.01) and London (Mean = -0.17, SD = 0.67). There was no significant difference t (df = 8.50) = 0.71, *p* = 0.5976. It is also important to note imbalance in the sample across locations, highlighted in the limitations section.
